# Recurrence Features and Factors influencing Post-relapse Survival in Early-stage Endometrial Cancer after Adjuvant Radiotherapy

**DOI:** 10.7150/jca.65246

**Published:** 2022-01-01

**Authors:** Kang Ren, Wenhui Wang, Shuai Sun, Dunhuang Wang, Xiaoliang Liu, Xiaorong Hou, Ke Hu, Fuquan Zhang

**Affiliations:** Department of Radiation Oncology, Peking Union Medical College Hospital, Chinese Academy of Medical Science & Peking Union Medical College, Beijing, China.; Xiaorong Hou, Ke Hu and Fuquan Zhang contributed equally to this work.

**Keywords:** endometrial cancer, post-recurrence survival, salvage treatment, reirradiation

## Abstract

**Purpose:** To evaluate the recurrent patterns and effect of clinicopathological factors on survival after recurrence (R-OS) in early stage endometrial cancer (EC).

**Methods:** Patients with FIGO stage I-II EC, who underwent post-surgery radiotherapy (RT) at our institution between 2000 and 2017, were enrolled. First recurrent patterns, overall survival (OS), and R-OS were evaluated. Univariate and multivariate analyses (MVA) were used to evaluate factors associated with R-OS.

**Results:** 756 patients were analyzed including 510 patients who received vaginal brachytherapy (VBT) and 246 patients who received external beam radiotherapy (EBRT) ± VBT, of whom 66 patients experienced recurrence, including 21 locoregional relapses and 45 distant metastases. Outside RT field recurrence predominated intra-RT field recurrence (106 versus 10 lesions). The 5-year OS rates for patients with and without recurrence were 62.2% and 98.2%, respectively (p<0.001). Among patients who underwent previous VBT, the 5-year OS rates were 61.1%, 92.3%, and 99.1% for distant metastasis, locoregional relapse, and non-recurrence, respectively (p<0.001); among patients who received EBRT ± VBT, the 5-year OS rates were 51.4%, 50.0%, and 98.3%, respectively (p<0.001).On Cox MVA of R-OS for locoregional recurrence patients, para-aortic lymph node metastasis was associated with poorer R-OS (hazard ratio [HR] 10.047, p=0.039), and salvage RT was superior to other therapies (HR 0.06, p=0.026). On Cox MVA of R-OS for distant metastasis, patients with brain metastasis (p=0.041) had the worst R-OS and patients benefited most from combined therapy (HR 0.02, p=0.001).

**Conclusion:** Recurrent patterns were dominated by outside RT field and distant metastasis for early-stage ECs after adjuvant RT. The modality of prior RT had an impact on the choice of salvage therapy. RT could still be an effective salvage treatment for patients who develop locoregional recurrence. Patients with distant metastasis may benefit from combined therapies.

## Introduction

Despite advances in the molecular pathology of endometrial cancer (EC), survival has not significantly improved in the past 30 years: the five-year survival rate was 83.18% in 2015 and 81.81% in 1985 according to the Surveillance, Epidemiology, and End Results (SEER) database 1, 2. Therefore, reducing the failure rate of post-therapy and improving the survival of ECs is the current focus 1. Radiotherapy (RT) is an important part of the adjuvant treatment regimens for EC, which reduces the locoregional recurrence and further improves survival 3. However, despite curative surgery and adjuvant RT received, approximately 15% of patients with early stage EC still develop recurrence 4.

Recurrence after curative treatment has a strong negative impact on survival 5, 6. Thus, improving survival after recurrence (R-OS) is another key to enhancing prognosis. R-OS is influenced by multiple factors including failure patterns, previous treatment, and salvage treatment 7. Previous studies demonstrated that patients with recurrence who did not receive previous RT had better R-OS 8. Lindemann et al. proposed RT for the salvage treatment of isolated pelvic relapse rather than the primary adjuvant regimen because the rate of salvage RT was higher in RT-naïve patients than those who received RT 9. Thus, the primary adjuvant treatment modality has a profound impact on salvage treatment choice. Although the type of adjuvant RT did not affect overall survival according to GOG 249 and PORTEC-2 10, 11, patients with locoregional recurrence who received prior RT, especially the pelvic radiation, may not be considered the reirradiation due to cumulative toxicities 6, 12. Furthermore, researches investigating the effectiveness of previous RT mode on R-OS and feasibility of reirradiation are still lacking.

Management options for recurrent patients include pelvic exenteration, RT/re-irradiation, chemotherapy, or the combination of them 13, 14. Decisions regarding salvage treatment regimens can be complicated because multiple factors should be considered including the relapse site and primary treatment, especially RT history. It remains challenging to achieve a balance between radical intent and tolerance of the normal organs in the setting of re-irradiation for intra-field recurrences 12, 14. To date, few studies have directly compared different salvage treatment regimens for recurrent early stage ECs in patients who underwent previous RT.

Considering the limited understanding of the management of recurrent EC, the present study aimed to clarify the first recurrence patterns in detail. We evaluated the clinicopathological and previous treatment-related characteristics associated with R-OS and analyzed the effectiveness of various salvage treatments categorized according to recurrence site to gain a comprehensive understanding of risk factor-outcome relationships of early stage ECs.

## Materials and methods

### Patient characteristics

The present study retrospectively reviewed the EC patients between Jan 2000 to Dec 2017 at Peking Union Medical College Hospital. The inclusion criteria for this study were as follows: diagnosis of stage I-II EC according to the 2009 International Federation of Gynecology and Obstetrics (FIGO); underwent surgery at Peking Union Medical College Hospital, including radical hysterectomy and unilateral or bilateral salpingo-oophorectomy with or without pelvic and para-aortic lymph node dissection; and received post-surgery adjuvant radiotherapy; with complete surgical pathology data and follow-up information. Individuals in whom surgical approaches were uncertain, those who did not complete adjuvant treatment, those with incomplete information regarding pathological and recurrence sites, and those with a follow-up < 6 months were excluded. Epidemiological data, previous and salvage treatment information, and follow-up records were collected and analyzed.

### Previous treatment regimen

The adjuvant treatment included EBRT, VBT, EBRT+VBT, and chemotherapy. The decision of adjuvant treatment was based on the pathological characteristics, mode of primary surgery, physical status, the willingness of patient, and discretion of doctors. The risk classification of the patient was redefined by the European Society for Medical Oncology (ESMO) - European Society of Gynecological Oncology (ESGO) - European Society for Radiotherapy & Oncology (ESTRO) 15.

EBRT alone was delivered with a total dose of 45 to 50.4 Gy in 23-28 fractions. The target volume of EBRT covered the region of the vaginal cuff, the proximal half of the vagina, and the pelvic lymph node drainage area. VBT only was administered using a high-dose-rate (HDR) iridium source (Ir-192) after a loading technique at 5 Gy per fraction within 5 to 6 fractions. For the group of EBRT + VBT, EBRT was administered with a total dose of 39.6Gy to 50.4Gy, and the following VBT was delivered at doses ranging from 4 to 6 Gy per fraction in 2 to 4 fractions. A vaginal cylinder was delivered 5 mm below the vaginal surface, and the target volume covered the vaginal cuff and proximal half of the vagina.

Patients received chemotherapy based on their clinicopathological factors and the discretion of their doctor. The chemotherapy regimens consisted of concurrent weekly regimen (cisplatin-based) or sequential three-weekly regimen (carboplatin/paclitaxel, intravenously).

### Recurrence patterns classified according to site and previous radiation field

Recurrence was evaluated by the clinical symptoms and signs, laboratory results, pathological biopsy if possible, and radiological examination results including abdominal, pelvic ultrasound, computed tomography (CT), magnetic resonance imaging (MRI), or positron emission tomography-CT (PET-CT). First recurrence events were categorized as locoregional recurrence or distant metastasis. Locoregional recurrence was defined as pelvic and regional recurrences including vaginal, pelvic, and para-aortic lymph nodes metastasis (PALM). Recurrences located beyond the area of pelvic and para-aortic and spread to the distant viscera and remote lymph nodes, including the lung, liver, bone, brain, inguinal lymph nodes, and the sus diaphragmatic lymph nodes, were categorized as distant metastases.

Locoregional recurrences were further classified as intra-RT field and outside RT field depending on whether or not recurrence lesions were located within the prior radiation field. For the patients who received previous pelvic EBRT ± VBT, recurrences located beyond the pelvic irradiated area were defined as outside RT field recurrence. For the patients who received previous VBT alone, recurrences located beyond the area of the vaginal cuff and proximal half of the vagina were defined as outside RT field recurrence.

### Salvage treatment and toxicity evaluation

Salvage treatment consisted of surgery, radiotherapy (RT), chemotherapy (CT), combined therapies (at least two types of treatment modalities such as RT plus CT, RT plus surgery, or the combination of RT, CT, and surgery), and other therapies (including endocrine treatment, palliative care).

Salvage RT consisted of VBT with or without EBRT or EBRT alone. EBRT was administered with intensity-modulated radiotherapy (IMRT) or tomotherapy. For patients with locoregional recurrences, EBRT was delivered at a dose of 45 to 50.4 Gy in 25 to 33 fractions, followed by a boost to the tumor region at the maximum dose of 20 Gy. Salvage VBT alone was administered with three-dimensional image-guided HDR Ir-192 after the loading technique of 30 Gy at 5 Gy per fraction.

Toxicities were evaluated according to the Common Terminology Criteria for Adverse Events (CTCAE) version 3.0.

### Statistical analysis

Overall survival (OS) was defined as the period from the date of primary surgery to the date of death or the last follow-up. R-OS for recurrent patients was defined as the period from the date of the first recurrence to the date of death or last follow-up. Disease-free interval (DFI) was defined as the interval between the initiation of treatment and the first relapse.

A Cox proportional hazards regression analysis was used to analyze the effectiveness of all parameters on R-OS. Univariate analysis (UVA) incorporated factors including age, risk groups, histological type, DFI, recurrence mode, and primary and salvage treatment regimens. Parameters with p values < 0.1 in the univariate analyses of the whole patients were included in the multivariate Cox proportional hazards model. Differences with p < 0.05 were considered to be statistically significant. Data were analyzed using SPSS version 26.0 (IBM Corporation, Armonk, NY, USA).

## Results

### Patient characteristics

**Figure 1:**
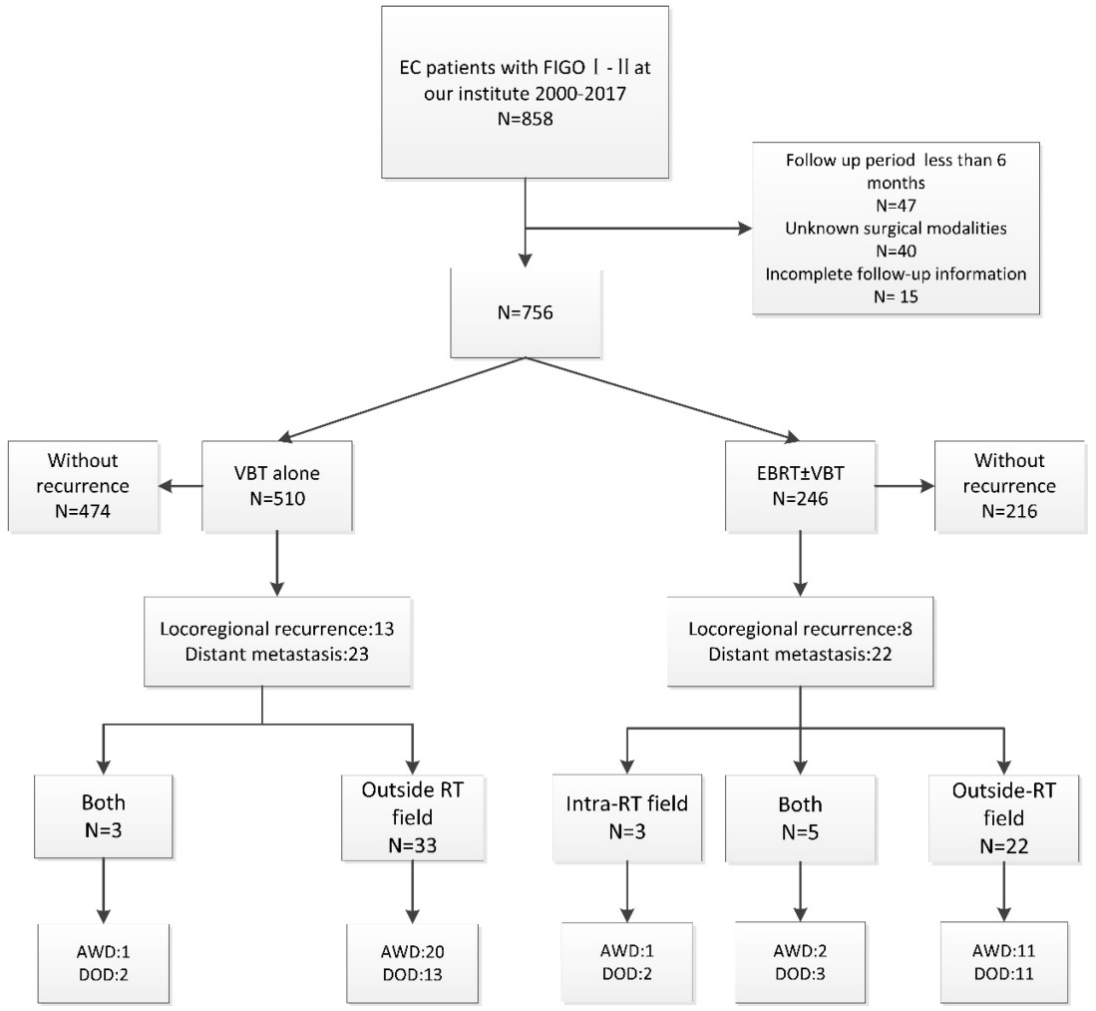
** The process and outcome of the study.** FIGO: International Federation of Gynecology and Obstetrics; VBT: vaginal brachytherapy; EBRT: external beam radiotherapy; Chemo: chemotherapy; AWD: alive without disease; DOD, died of disease.

A total of 756 patients were analyzed with the median follow-up period of 61 months, 66/756 (8.7%) of patients experienced recurrences. 21/66 (31.8%) patients experienced locoregional recurrence only and 45/66 (68.2%) patients developed distant metastasis including 6 patients with simultaneous PALM, 6 patients with simultaneous vaginal or pelvic recurrences. Additionally, 10.6% (7/66) of patients died after locoregional recurrence, and 47.0% (31/66) died due to distant metastasis. **Figure 1** illustrated the procedure for the analysis approach of the study and patients' outcomes.

Compared to patients without recurrence, those who experienced recurrence were more often > 65 years old (p = 0.010), had a higher pathological grade (p < 0.001), higher FIGO stage (p < 0.001), and more commonly classified as high-intermediate risk or high risk groups (p < 0.001) (**Table 1**).

### Details of recurrence features

**Table 2** showed the distribution of recurrent lesions according to the prior RT field. Outside RT field recurrence was the predominant recurrence pattern over the intra-RT field recurrence (106 vs 10 lesions). Among the 36 recurrent patients who received prior VBT alone, three recurrent lesions were located in the stump or the wall of the vagina within the irradiation field and 60 lesions located beyond the field area. The most common distant metastasis site located outside RT field was the lung.

Among patients who received prior EBRT ± VBT, 30 patients experienced recurrence. Out of the 7 lesions located within the radiation field, the pelvic or pelvic lymph nodes (6/7) were the most common recurrence site, followed by the vaginal stump (1/7). Among the 46 outside RT field lesions, lungs were the most frequent recurrent site (10/46) and the brain metastases (6/46) were more common than the patients who received prior VBT. Seven lesions were located in the para-aortic lymph node drainage area. Of note, a total of ten vaginal lesions were located in the lower segment of the vagina beyond the irradiation field.

### Survival outcome

**Figure 2:**
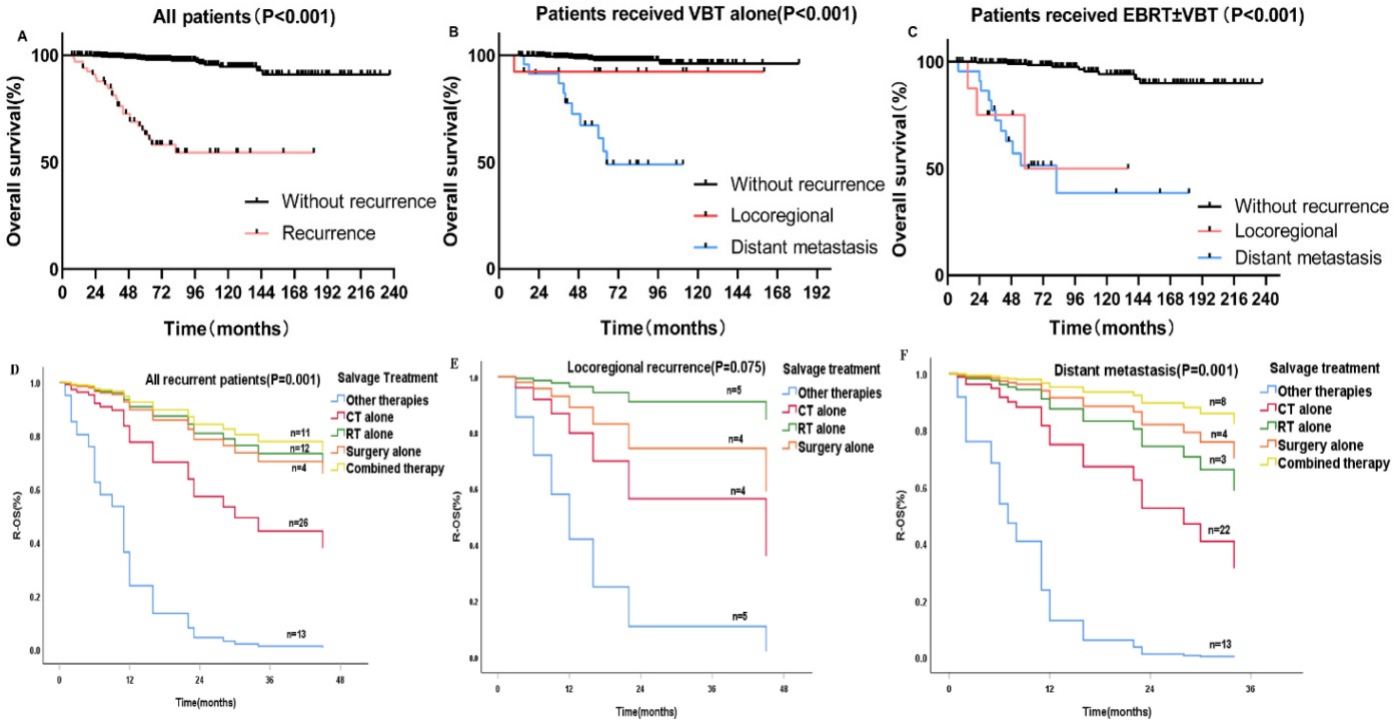
** A-C** Kaplan-Meier survival analysis of the OS in all and subgroups of patients. **D-F** Cox regression model of R-OS in all and subgroups of recurrent patients: (A) OS in the whole cohort according to patients with and without recurrence. (B) OS in the VBT group according to patients with and without recurrence. (C) OS in the EBRT±VBT group according to patients with and without recurrence. (D) R-OS in all of the recurrent patients categorized by various salvage therapies. (E) R-OS in locoregional patients. (F) R-OS in distant metastasis patients.

The 5-year OS for patients with and without recurrence were 62.8% and 97.9%, respectively (p < 0.001) (**Figure 2A**). For patients who underwent previous VBT alone, the 5-year OS rates for distant metastasis, locoregional relapse, and non-recurrence patients were 61.1%, 92.3%, and 97.7%, respectively (p < 0.001) (**Figure 2B**). For patients who received previous EBRT ± VBT, the 5-year OS rates for distant metastasis, locoregional relapse, and non-recurrence were 51.4%, 50.0%, and 99.5%, respectively (p < 0.001) (**Figure 2C**).

For the patients with recurrence, the median R-OS was 45 months. The 1-, 2-, and 3-year R-OS rates were 74.8%, 61%, and 53.4%, respectively. For patients with locoregional recurrence, the 3-year R-OS for patients who received previous EBRT ± VBT and VBT alone were 29.2% and 80%,respectively (p<0.05). For patients with distant metastasis, the 5-year R-OS of patients who received previous EBRT ± VBT were 35.9% and 52.7% (p=0.671) (Supplementary Figure 2).

### Details of the salvage treatment

Among patients with locoregional recurrence (N=21), eight underwent previous EBRT, and 13 received previous VBT alone. Of the eight patients who received previous EBRT, 25% (2/8) received re-irradiation alone, 25% (2/8) received chemotherapy alone, and 12.5% (1/8) received a combination of radiotherapy and resection surgery. One patient who received salvage RT, with the shortest radiation interval of 7 months, experienced grade 3 gastrointestinal toxicity and died of tumor progression. The remaining two patients, who received salvage RT, were alive without progression during follow-up (**Table 3**).

Among the patients who received previous VBT alone, 46.2% (6/13) underwent salvage RT, 23.1% (3/13) underwent a combination of RT and surgery, and 15.4% (2/13) received chemotherapy alone. None of the patients who received salvage radiation or the combined therapies experienced grade ≥ 3 toxicities, and all of these patients were alive without tumor progression (**Table 3**). The median time interval of the re-irradiation was 25 months (range: 7 to 120 months). The median re-irradiation EBRT dose was 50.4Gy (range: 45 to 70.4Gy) in 25 to 33 fractions, and the re-irradiation VBT dose was 30Gy in 6 fractions (Supplementary Table 1).

Among patients with distant metastasis (N=45), two patients received salvage RT, 22 patients received chemotherapy alone, four patients underwent surgery, eight patients were administrated with combined therapies and eight patients received other salvage therapies. 15 patients developed G1-2 toxicities and none of the patients experienced grade > 3 toxicities.

### Cox regression analyses of R-OS

For all patients who experienced recurrence (N=66), ≥65 years old (p = 0.003), higher pathological grade (p = 0.025), PALM (p = 0.023), DFI < 12 months (p = 0.022) and salvage treatments other than RT, CT, or surgery (p < 0.001) were associated with poorer R-OS. On multivariate Cox analysis, only PALM (HR 3.19, p = 0.012) and salvage treatment (p = 0.001) were independent factors for R-OS (**Table 1**).

For patients with locoregional recurrence(s), PALM (p = 0.042), previous treatment (p = 0.043), previous RT mode (p = 0.032), and salvage treatment (p = 0.029) were associated with R-OS. On multivariate Cox regression analysis, PALM (HR 3.19, p = 0.012) was significantly associated with poorer R-OS than in those without PALM (**Table 1**). Patients who received salvage RT alone (HR 0.06, p = 0.026) had a better R-OS prognosis than those who received other therapies with other therapy as a reference (**Figure 2E**).

For patients with distant metastasis, Cox univariate analysis revealed that age (p = 0.021), metastatic site(s) (p = 0.021), and salvage treatment (p = 0.001) were associated with R-OS. On Cox multivariate regression modeling, the combined treatment regimen improved survival more than other entities with other therapy as a reference (HR 0.024, p = 0.001) (**Figure 2F**). And patients with brain metastasis had the worst R-OS than patients with other sites of metastasis (p=0.041).

### Subgroup analysis of patients with PALM

Among the 14 patients who experienced PALM, six developed distant metastasis, six had isolated PALM, and two experienced concomitant pelvic lymph node metastasis. Four PALM patients presented with abdominal pain, and five with PALM were asymptomatic and diagnosed using CT or PET/CT. Compared with low-risk patients, PALM was more common in the high- and intermediate-risk groups (p = 0.04) (Supplementary Table 2). The 3-year R-OS rates for patients with and without PALM were 34.3% and 56.9%, respectively (p = 0.016) (Supplementary Figure 1). The result of Cox multivariate revealed that only salvage RT was a significant predictor of R-OS (HR 0.02, p = 0.024) (Supplementary Table 3).

## Discussion

Our study elucidated the recurrence features of early stage ECs received adjuvant radiotherapy. We found that outside RT field recurrences predominated over the intra-RT field recurrences and distant metastasis was the majority recurrence pattern. The prior radiotherapy modalities have different impacts on the choice of salvage treatment and influenced subsequent outcomes after recurrence. Furthermore, multivariate analysis indicated that the salvage regimen was the independent predictor for R-OS. RT remained the most optimal salvage treatment for locoregional recurrence with a low incidence of serious toxicities.

Patients who experienced recurrence had significantly poorer outcomes with 5-year OS decreasing to 62.8% in our study. These results were comparable to previous studies that Samual R. Francis et al. reported the 5-year OS rate for early stage ECs with recurrence was 58.3% with the median OS of 46.8 months 4. The present study reaffirmed that distant metastasis was the predominant recurrence pattern for patients who received prior RT, the failure pattern was different from the patients who received surgery only, for whom local recurrence including the vaginal vault and nodal rather than distant metastasis is the main recurrent sites 16, 17. The discrepancy may be due to the addition of adjuvant RT reducing the rate of vaginal and pelvic relapse 6, 18, the same finding was previously raised by Carien L. Creutzberg et al. that distant metastasis was the most recurrence pattern for the RT group while the vaginal recurrences were more common in the patients without previous RT 8. Notably, the result revealed that most outside RT field recurrences were not accompanied by the intra-field recurrence, suggesting that outside RT field relapse maybe not develop secondary to local failure and distant micro-metastases possibly existed before RT 19, thus highlighting the importance of systemic treatment 20.

The present study also explored the effectiveness of the first course of treatment modalities on survival outcomes, and the result indicated that R-OS was better in locoregional patients who received prior VBT alone than EBRT ± VBT (p<0.05). Further, Cox UVA revealed that the prior VBT alone was associated with improved R-OS (HR 0.094, p=0.032) but lost independent significance on Cox MVA. Specifically, the salvage re-irradiation rate of the VBT group was 69.2% (9/13) but decreased to only 37.5% (3/8) in the EBRT ± VBT group.

A similar observation was also reported by a follow-up analysis of the PORTEC-1 trial, with a salvage radiation rate of 93.8% (30/32) in RT-naïve patients, which decreased to 71.4% (5/7) in patients who underwent prior radiation 8. Generally, patients who received previous pelvic radiation would not be considered for radiation again, in particular the salvage EBRT, and patients who received VBT alone can be treated as “RT-naïve” according to the National Comprehensive Cancer Network (NCCN) guidelines 21. The decreased rate of salvage re-irradiation may account for decreased survival outcomes since RT has been demonstrated to be the effective method for recurrent tumors 14, 22, 23.

Salvage treatment regimens consisted of pelvic exenteration, re-irradiation, or systemic chemotherapy, etc. 13, 21. However, heterogeneity remains high even in the population of patients with locoregional recurrence, which makes it difficult to reach a consensus on the treatment recommendations. Our study demonstrated that for patients with locoregional recurrence, salvage RT (HR 0.051, p = 0.026) resulted in the best survival outcomes compared with a combined regimen, chemotherapy alone, and other treatment. In accordance with the present results, Francis SR et al. demonstrated that RT was the only significant factor of prognosis for vaginal-only recurrent patients, but patients with pelvic recurrence benefited more from multimodal treatment. But only 25% of the enrolled patients received adjuvant RT, and the proportion of patients undergoing re-irradiation was unclear in their study 4. In addition, Li Lei et al. found the salvage RT led to the best OS for recurrent cervical cancer within the pelvic cavity compared with other therapies 24.

Currently, there is no specific consensus on the doses, modalities, and time intervals of re-irradiation. Researches in re-irradiation for EC with locoregional recurrences have remained rather limited. We administrated salvage re-irradiation with the median dose of 50.4Gy for EBRT and 30Gy for VBT, and the median re-irradiation interval was 25 months. The results revealed that only one patient with a short re-irradiation interval experienced grade 3 toxicity. The re-irradiation regimen for locoregional recurrences in our center was similar to those reported in previous studies. The range of salvage radiation dose reported in the study of Ling, D. C.et al. was 45.0 Gy (range: 24-45) for EBRT and 28.8 Gy (range: 23.4-30.6 Gy) for 3D conformal VBT with an interval of re-irradiation of more than 20 months 16. Another study conducted by H.Raziee et al. adopted interstitial brachytherapy (ISBT) to treat pelvic recurrences with the median dose of 29.1 Gy (range 16.1-64.6) and median re-irradiation interval of 20.3 months 25. None of these studies researches reported serious toxicities. It seems that re-irradiation delivered by IMRT or 3D VBT can serve as a safe and effective salvage treatment means for locoregional recurrence patients who received prior RT.

The present results indicated that PALM was an independent predictor of poor R-OS in patients with locoregional recurrence (HR 10.047, p=0.039). If PALM is detected before distant metastases, patients will have a higher chance of being cured 26. Our study revealed that the high-intermediate and high-risk patients were more susceptible to PALM metastasis (p<0.05), highlighting the importance of regular abdominal screening in the follow-up for this group of patients. Only salvage RT was significantly associated with improved survival (HR 0.02, p=0.024) for PALM with only one patient experiencing grade 3 hematological toxicity in our study. Due to the special anatomic location, it was hard to resect the tumor completely by the surgery alone. Recently, an increasing number of studies have affirmed the effect of RT on treating gynecological cancer patients with PALM. Shirvani, S. M et al. reported a grade 3-5 gastrointestinal toxicity rate of 19%, they adopted the IMRT technique to treat PALM 26. While a study by Diane C Ling et al. showed a lower grade ≥3 toxicity rate of 14.3% by using the stereotactic body (SBRT) 27. Prospective studies are warranted to explore radiation modality and doses to further attenuate toxicities.

For patients with distant metastasis, we found that site-specific metastasis patterns had different effects on survival, with the poorest prognosis for brain metastasis and relatively better survival for liver and lung metastases. These observations were in agreement with Ouldamer L's finding which showed that the most common site of metastasis was the lung and brain metastatic disease has a shorter 3-year OS than lung or other site metastasis 20. In the present study, a multimodal treatment regimen consisting of a combination of surgery and chemotherapy or radiation and chemotherapy was demonstrated to be more effective. Of note, patients who underwent salvage surgery alone experienced superior survival outcomes compared with radiation alone, which was consistent with the results of a previous study 28, suggesting that the feasibility of the second surgical resection, especially for solitary metastasis, is an important factor for improved survival.

To our knowledge, literature addressing recurrence patterns and outcomes for patients receiving RT is sparse. Molecular subgroups of EC result in potential novel treatment strategies for patients with recurrence 29. For example, recurrence patients with POLEmut or MMRd may benefit from immunotherapy such as anti-PD1 immune checkpoint blockade 30. p53mut ECs are the group with the high risk of recurrence and worst prognosis. Some potential molecular targets are identified in the p53mut group such as amplification of the ERBB2 gene, which may serve as a therapeutic target for recurrent patients 29, 31. The results of PORTEC-4a for better selecting adjuvant treatment for ECs using molecular classifications have not yet been published and are highly expected 32. The molecular risk classifications subjected to detecting the POLE sequencing and other immunohistochemical parameters cannot readily be generalized to clinical practice. As such, this study contributes evidence to the decision-making process for different salvage treatment regimens especially in the absence of molecular results.

However, there are also limitations to our study, the first of which is its retrospective design and the relatively small number of recurrent patients, which precluded us from drawing definitive conclusions. Secondly, we did not identify which salvage treatment regimen prolonged survival after recurrence in patients with pelvic recurrence, vaginal recurrence, and PALM, most probably because the number of patients with recurrence was not sufficient to further stratify according to recurrence patterns.

## Conclusion

To further improve the prognosis of early stage ECs, a comprehensive understanding of the recurrent patterns and relationship between treatment-related factors and survival outcomes is of great significance. The choices of salvage treatment were affected by recurrent patterns and the modality of prior RT. Multivariate analyses showed that salvage RT can be chosen as the optimal treatment option even for the previously irradiated patients with locoregional recurrences. Multimodal treatment may be more effective for patients with distant metastasis.

## Supplementary Material

Supplementary figures and tables.

## Figures and Tables

**Table 1:** Patient and tumor characteristics

Characteristics	Whole patients (n=756)	%	Without recurrence (n=690)	%	Recurrence (n=66)	%	p
**Age (years)**							
<65	654	86.50%	604	87.50%	50	75.80%	**0.01**
≥65	102	13.50%	86	12.50%	16	24.20%	
**Pathological type**							
Type 1	716	94.70%	655	94.90%	61	92.40%	0.383
Type 2	40	5.30%	35	5.10%	5	7.60%	
Grade†							
1	308	40.70%	296	42.90%	12	18.20%	**<0.001**
2	317	41.90%	286	41.40%	31	47.00%	
3	109	14.40%	90	13.00%	19	28.80%	
LVSI							
Yes	135	17.90%	120	17.40%	15	22.70%	0.312
No	621	82.10%	570	82.60%	51	77.30%	
**FIGO stage**							
Ia	410	54.20%	383	55.50%	27	40.90%	**<0.001**
Ib	304	40.20%	276	40%	28	42.40%	
II	42	5.60%	31	4.50%	11	16.70%	
**Risk stratification**							
Low risk	272	36.00%	259	37.50%	13	19.70%	**<0.001**
Intermediate risk	220	29.10%	204	29.60%	16	24.20%	
High-intermediate risk	155	20.50%	137	19.90%	18	27.30%	
High risk	109	14.40%	90	13.00%	19	28.80%	
Prior treatment regimen							**<0.001**
VBT alone	481	63.60%	449	65.10%	32	48.50%	
EBRT ± VBT	187	24.70%	169	24.50%	18	27.30%	
VBT + chemo	29	3.80%	25	3.60%	4	6.10%	
EBRT ± VBT+ chemo	59	7.80%	47	6.80%	12	18.20%	

Abbreviations: EEC: endometrioid endometrial carcinoma; LVSI, lymph-vascular space invasion. Chemo: chemotherapy. FIGO: International Federation of Gynecology and Obstetrics; VBT: vaginal brachytherapy; EBRT: external beam radiotherapy; chemo: chemotherapy. Note: Grade†: Non-endometrioid endometrial types were excluded from this group. Bold indicates p-Value <0.05, considered significant.

**Table 2:** Distribution of the recurrent lesions according to the RT modality

Recurrent site	VBT (36 recurrence patients)		EBRT (30 recurrence patients)	
	Intra-field lesions	Outside field lesions	Intra-field lesions	Outside field lesions
Stump and upper of the vagina	3		1	
Lower vagina		5		5
Pelvic/LN		7	6	
Para-aortic LN		7		7
Lung		22		10
Peritoneal		6		5
Liver		5		5
Bladder		2		1
Extraperitoneal cavity LN		3		5
Bone		3		2
Brain		0		6
**Total**	**3**	**60**	**7**	**46**

**Table 3:** Details of salvage treatment according to recurrent patterns and prior RT modes

	Locoregional (n=21)			Distant metastasis (n=45)		
	VBT (n=13)	EBRT±VBT (n=8)	Total	VBT (n=23)	EBRT±VBT (n=22)	Total
**Salvage treatment**						
Other therapies	2	3	5	5	3	8
RT	6	2	8	2	1	3
CT	2	2	4	11	11	22
Surgery	0	0	0	3	1	4
**Combined therapy**						
Surgery + RT	2	1	3	0	0	0
RT+CT	0	0	0	1	3	4
CT±RT+Surgery	1	0	1	1	3	4
Total	3	1	4	2	6	8
**Toxicities**						
G1-G2	1	2	3	4	11	15
≥G3	0	1	1	0	0	0
**Treatment results**						
Alive without death	11	3	14	13	11	24
Dead of EC	2	5	7	10	11	21

Abbreviations: FIGO, International Federation of Gynecology and Obstetrics; VBT, vaginal brachytherapy; EBRT, external beam radiotherapy; chemo, chemotherapy. G, grade.

**Table 4:** Multivariate Cox regression analysis for R-OS

	All patients			Locoregional recurrence			Distant metastasis		
	HR	95% CI	p	HR	95% CI	p*	HR	95% CI	p†
**Age (years)**									
<65	Ref								
≥65	2.361	1.019-5.47	**0.045**						
**Grade**									
G1	Ref								
G2	0.23	0.042-1.257	0.09						
G3	2.211	0.356-13.718	0.394						
**Risk stratification**									
LR	Ref		0.189						
IR	3.391	0.696-16.527	0.131						
HIR	0.534	0.112-2.542	0.43						
HR	0.469	0.075-2.949	0.42						
**DFI (months)**									
<12	Ref								
≥12	1.206	0.347-4.187	0.768						
**PALM**									
Negative	Ref			Ref		**0.039**			
Metastasis	3.19	1.294-7.861	**0.012**	10.047	1.128-89.482				
**Metastasis sites**									
Brain							Ref		**0.041**
Lung							0.081	0.014-0.469	**0.005**
Liver							0.056	0.005-0.686	**0.024**
Other							0.084	0.013-0.525	**0.008**
Multiple							0.184	0.035-0.957	**0.044**
Bone							0.061	0.005-0.691	**0.024**
**Prior RT mode**									
VBT	Ref		0.099						
EBRT±VBT	0.444	0.169-1.166							
**Salvage treatment**									
Other therapies	Ref		**<0.001**	Ref		0.075	Ref		0.001
CT	0.076	0.02-0.287	**<0.001**	0.913	0.089-9.407	0.939	0.131	0.038-0.455	**0.001**
RT	0.028	0.004-0.177	**<0.001**	0.051	0.004-0.703	**0.026**	0.049	0.005-0.516	**0.012**
Surgery	0.024	0.002-0.36	**0.007**	—	—	—	0.048	0.004-0.591	**0.018**
Combined therapy	0.009	0.001-0.084	**<0.001**	0.083	0.006-1.088	0.058	0.02	0.002-0.202	**0.001**

Bold indicates p-Value <0.05, considered significant; p * and p †: only the factors with the p-value<0.05 in the multivariate regression analysis were shown in the table; Abbreviations: Ref, reference.LR, Low risk.IR, Intermediate risk. HIR, High-intermediate risk.HR, high risk. G: pathology grade; HR: hazard ratio; CI: confidence interval; RT: radiotherapy; CT: chemotherapy; R-OS: overall survival after recurrence.
