# Supplementary material for: Recurrence Features and Factors influencing Post-relapse Survival in Early-stage Endometrial Cancer after Adjuvant Radiotherapy
Source: J Cancer. 2022 Jan 1;13(1):202–11. doi: 10.7150/jca.65246 (PMC8692693; doi:10.7150/jca.65246)
Supplement: Supplementary file 1 — Supplementary figures and tables. [file jcav13p0202s1.pdf]

Table S1. Details of salvage re-irradiation for locoregional recurrence

Prior RT type	Age	First relapse site	Relapse pattern	Salvage treatment	Reirradiation technique	Median reirradiation interval (months)	Salvage RT doses	Outcomes	Toxicities
VBT	58	Vagina	Intra-RT field	EBRT	Unclear	26	Unclear	AWD	Non
	63	Vagina	Both	Surgery +VBT	3D VBT	33	HRCTV:30Gy/6f	AWD	G2 proctitis
	61	Vagina/ pelvic	Both	EBRT+VBT	IMRT+3DVBT	37	PGTV:60.2Gy/28f, PCTV:50.4Gy/28f, HRCTV:30Gy/6f	AWD	Non
	34	Pelvic	Outside RT field	EBRT	Unclear	40	Unclear	AWD	Non
	67	Pelvic	Outside RT field	EBRT	IMRT	17	PCTV:50.4Gy/28f, PGTV:61.2G/28f	AWD	Non
	42	Pelvic	Outside RT field	EBRT	IMRT	40	PCTV:50.4Gy/28f	AWD	Non
	51	Vagina	Intra-RT field	VBT	2D VBT	9	30Gy/6f	AWD	G1 proctitis
	32	Pelvic /PALM	Outside RT field	EBRT	Unclear	23	Unclear	AWD	Non
	62	PALM	Outside RT field	EBRT	IMRT	24	PCTV:50.4Gy/28f	AWD	Non
EBRT+VBT	62	Vaginal	Intra-RT field	EBRT	TOMO	120	PCTV:70.4Gy/33f	AWD	G2 mucous toxicity
	57	PALM	Outside RT field	EBRT	IMRT	20	PCTV:45Gy/25f, PGTV:62.5Gy/25f	AWD	Non
	79	PALM	Outside RT field	EBRT	TOMO	7	PCTV： 50Gy/20f PGTV： 60Gy/20f	DOD	G3 haematological toxicity and G2 gastrointestinal toxicity

AWD: alive without disease. DOD: died of disease.

Table S2. The distribution of PALM according to the ESMO-ESGO-ESTRO risk classification

PALN	ESMO-ESGO-ESTRO risk classes				p
	Low risk	Intermediate risk	High intermediate risk	High risk	
Non-recurrence	12(92.3%)	14(87.5%)	10(55.6%)	16(84.2%)	**0.04**
Recurrence	1(7.7%)	2(12.5%)	8(44.4%)	3(15.8%)	

ESMO-ESGO-ESTRO: European Society for Medical Oncology (ESMO)–European Society of Gynecological Oncology (ESGO) – European Society for Radiotherapy & Oncology (ESTRO)

Table S3.Independent risk factors of Cox regression analysis for on survival after recurrence in PALM patients group

	Multivariate Cox Regression			
	HR	95% CI		p
Age（years）				
<65	Reference			0.101
≥65	5.314	0.722	39.114	
Prior radiotherapy modes				
VBT	0.637	0.096	4.231	0.641
EBRT±VBT	Reference			
Failure pattern				
Isolated PLAM	Reference			0.423
With pelvic recurrence	4.454	0.226	87.72	0.326
With distant metastasis	0.429	0.081	2.285	0.322
Salvage treatment				
RT	0.02	0.001	0.595	**0.024**
without RT	Reference			

FIGO: International Federation of Gynecology and Obstetrics; VBT: vaginal brachytherapy; EBRT: external beam radiotherapy; PALM: para-aortic lymph nodes metastasis

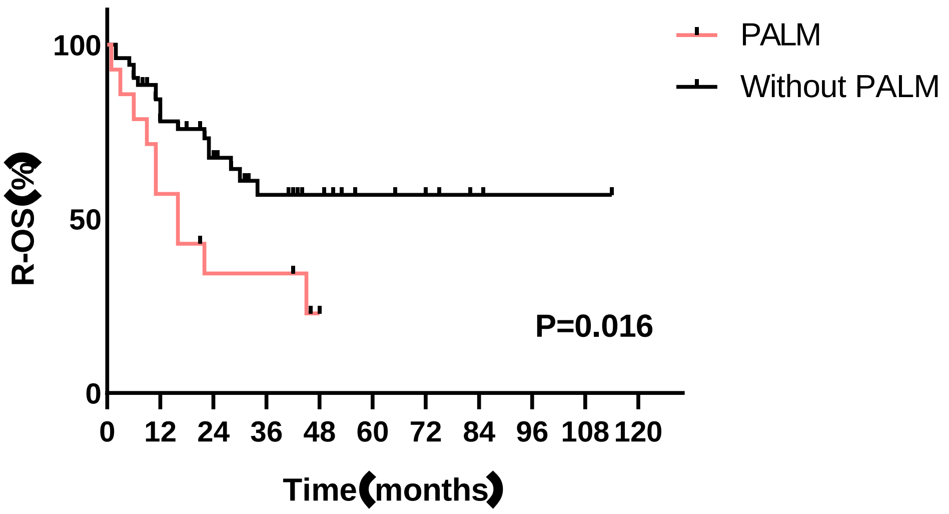

Figure S1. Kaplan-Meier estimates of survival after recurrence (R-OS) according to the status of para-aortic lymph node.

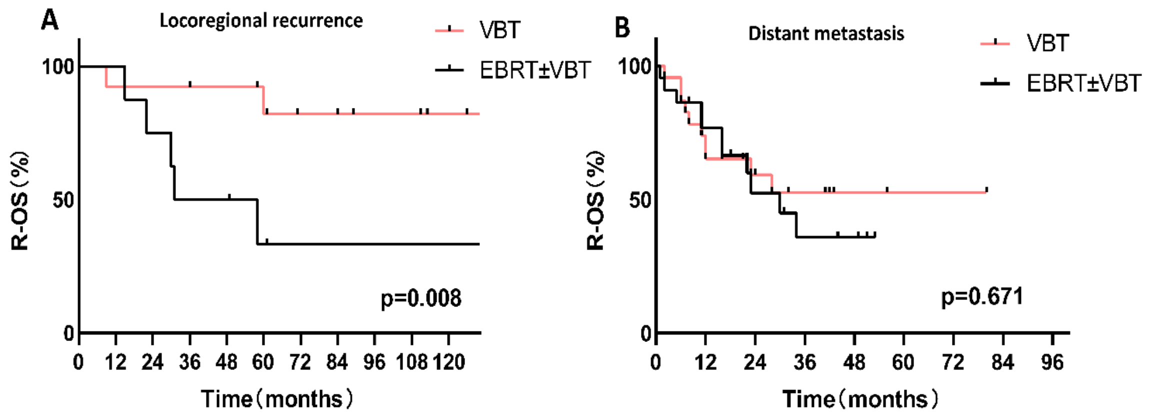

Figure S2. Kaplan-Meier estimates of survival after recurrence (R-OS). (A) R-OS for locoregional recurrence patients categorized by prior RT mode. (B) R-OS for distant metastasis patients categorized by prior RT mode.
